# A Histone-Like Nucleoid Structuring Protein Regulates Several Virulence Traits in Burkholderia multivorans

**DOI:** 10.1128/AEM.00369-21

**Published:** 2021-06-25

**Authors:** Sara C. Gomes, Mirela R. Ferreira, Andreia F. Tavares, Inês N. Silva, Jörg D. Becker, Leonilde M. Moreira

**Affiliations:** a Institute for Bioengineering and Biosciences, Instituto Superior Técnico, Lisbon, Portugal; b Instituto Gulbenkian de Ciência, Oeiras, Portugal; c Instituto de Tecnologia Química e Biológica António Xavier, Universidade Nova de Lisboa, Oeiras, Portugal; d Department of Bioengineering, Instituto Superior Técnico, Lisbon, Portugal; University of Tartu

**Keywords:** H-NS, exopolysaccharide, *Burkholderia*, virulence

## Abstract

Burkholderia cepacia complex bacteria comprise opportunistic pathogens causing chronic respiratory infections in cystic fibrosis (CF) patients. These microorganisms produce an exopolysaccharide named cepacian, which is considered a virulence determinant. To find genes implicated in the regulation of cepacian biosynthesis, we characterized an evolved nonmucoid variant (17616nmv) derived from the ancestor, Burkholderia multivorans ATCC 17616, after prolonged stationary phase. Lack of cepacian biosynthesis was correlated with downregulation of the expression of *bce* genes implicated in its biosynthesis. Furthermore, genome sequencing of the variant identified the transposition of the mobile element IS*406* upstream of the coding sequence of an *hns*-like gene (Bmul_0158) encoding a histone-like nucleoid structuring (H-NS) protein, a known global transcriptional repressor. This insertion sequence (IS) element upregulated the expression of Bmul_0158 by 4-fold. Transcriptome analysis identified the global effects of this mutation on gene expression, with major changes in genes implicated in motility, pilus synthesis, type VI secretion, and chromosome-associated functions. Concomitant with these differences, the nonmucoid variant displays reduced adherence to a CF lung bronchial cell line and reduced surface hydrophobicity and forms smaller cellular aggregates but has an increase in swimming and swarming motilities. Finally, analysis of the GC content of the upstream region of differentially expressed genes led to the identification of various genomic regions, possibly acquired by horizontal gene transfer, which were transcriptionally repressed by the increased expression of the Bmul_0158 gene in the 17616nmv strain. Taken together, the results revealed a significant role for this H-NS protein in the regulation of *B. multivorans* persistence- and virulence-associated genes.

**IMPORTANCE** Members of the histone-like nucleoid structuring (H-NS) family of proteins, present in many bacteria, are important global regulators of gene expression. Many of the regulated genes were acquired horizontally and include pathogenicity islands and prophages, among others. Additionally, H-NS can play a structural role by bridging and compacting DNA, fulfilling a crucial role in cell physiology. Several virulence phenotypes have been frequently identified in several bacteria as dependent on H-NS activity. Here, we describe an H-NS-like protein of the opportunistic pathogen Burkholderia multivorans, a species commonly infecting the respiratory tract of cystic fibrosis patients. Our results indicate that this protein is involved in regulating virulence traits such as exopolysaccharide biosynthesis, adhesion to biotic surfaces, cellular aggregation, and motility. Furthermore, this H-NS-like protein is one out of eight orthologs present in the *B. multivorans* ATCC 17616 genome, posing relevant questions to be investigated on how these proteins coordinate the expression of virulence traits.

## INTRODUCTION

Bacteria of the Burkholderia cepacia complex (Bcc) are a group of opportunistic pathogens affecting mostly cystic fibrosis (CF) patients but also immunocompromised persons. The predominant Bcc species isolated from infected CF patients’ lungs are Burkholderia multivorans and Burkholderia cenocepacia (1). These microorganisms often cause chronic respiratory infections, very difficult to eradicate due to their intrinsic antibiotic resistance, leading to progressive lung function deterioration (2). In a small percentage of CF patients, these infections progress to rapidly deteriorating pneumonia, followed by bacteremia and patient death. *Burkholderia* strains have large genomes (6 to 10 Mbp) consisting of three chromosomes and several plasmids (3, 4). This high number of genes, in combination with genome plasticity due to the presence of insertion sequences (ISs), transposons, and other mobile elements, makes these organisms highly adapted to very different environments, including eukaryotic hosts (3).

Various molecular mechanisms contributing to the virulence potential of Bcc have been identified, including type III and type VI secretion systems, extracellular enzymes, adhesins, fimbriae, siderophores, lipopolysaccharide, extracellular polysaccharides (EPS), and quorum sensing systems, among others (4–6). Among these determinants of pathogenicity, the expression of the mucoid phenotype due to the production of exopolysaccharide is of particular relevance, as this polymer is likely important to protect bacteria against the host immune system and antimicrobial compounds (7). In early-stage infections of CF airways caused by environmental strains, Bcc bacteria have the ability to produce extracellular carbohydrate polymers, but when infection progresses and lung function deteriorates, bacteria often lose this ability and become nonmucoid (7). Moreover, a negative correlation between the mucoid phenotype of clinical isolates and lung function deterioration has been shown (8). This suggests that EPS production might be important in Bcc colonization and persistence in the CF patients’ airways, but perhaps not at later stages of the infection. The EPS responsible for the mucoid phenotype, named cepacian, has been extensively characterized regarding its biosynthetic steps in *B. multivorans* and Burkholderia contaminans (9–11). Although the regulation of *bce* genes’ expression directing cepacian biosynthesis is not yet fully understood, the NtrBC signal transduction system and the alternative sigma factor σ^54^ have been implicated in regulating their expression in B. cenocepacia H111. In *B. multivorans*, another signal transduction system, EnvZ/OmpR, was identified as responsible for EPS biosynthesis, as nonmucoid colonies obtained under stress conditions accumulated mutations in the *ompR* gene (12, 13). The possible involvement of small RNAs (sRNAs) in cepacian biosynthesis regulation comes from the observation that a mutant in the *B. contaminans* IST408 RNA chaperone Hfq produces 50% less EPS than the wild-type strain, although no specific sRNA has been identified (14).

Another mechanism regulating EPS production in some Gram-negative bacteria, such as *Enterobacteria*, is mediated by the histone-like nucleoid structuring protein (H-NS). Proteins of this family function as architectural components of the nucleoid and as global regulators of gene expression (15). H-NS proteins are known to directly repress AT-rich foreign DNA acquired by horizontal gene transfer, facilitating tolerance and integration of these sequences into preexisting regulatory networks (16). Mutations in *hns*-like genes cause pleiotropic effects in several species, including changes in growth, motility, biofilm formation, antimicrobial resistance, adhesion to eukaryotic cells, and polysaccharide biosynthesis, among others. Regarding polysaccharide biosynthesis, an *hns* deletion mutant of Klebsiella pneumoniae increased capsular polysaccharide production, conferring a highly mucoid phenotype to colonies (17). Also, in Actinobacillus pleuropneumoniae and Vibrio cholerae, H-NS acts as a repressor of EPS biosynthesis genes (18, 19). Contrastingly, in Vibrio parahaemolyticus, H-NS acts as an activator of EPS biosynthesis gene transcription (20). Whether H-NS-dependent mechanisms also regulate EPS biosynthesis in *Burkholderia* has not yet been reported. In contrast to some *Enterobacteria*, *Burkholderia* strains are known to possess multiple *hns*-like genes within each genome, which remain mostly uncharacterized. The sole evidence of the role of an *hns*-like gene (BCAL0154) in virulence comes from a study in B. cenocepacia J2315, in which its deletion reduced bacterial accumulation and attenuated virulence in Caenorhabditis elegans (21).

This study reports the comprehensive characterization of a nonmucoid derivative of *B. multivorans* strain ATCC 17616. Whole-genome sequencing of this nonmucoid strain revealed the transposition of an insertion sequence (IS*406*) into an intergenic region, which caused increased expression of a gene encoding a protein from the H-NS family. Besides the changes observed to the mucoid phenotype, the mutant strain also displayed an altered adherence phenotype, as well as increased motility. In summary, this study describes the importance of a protein of the H-NS family in regulating the expression of *B. multivorans* persistence and virulence genes.

## RESULTS

### Reduction of the mucoid phenotype of a *B. multivorans* ATCC 17616 evolved colony pairs with the decreased expression of the *bce* genes.

**FIG 1:**
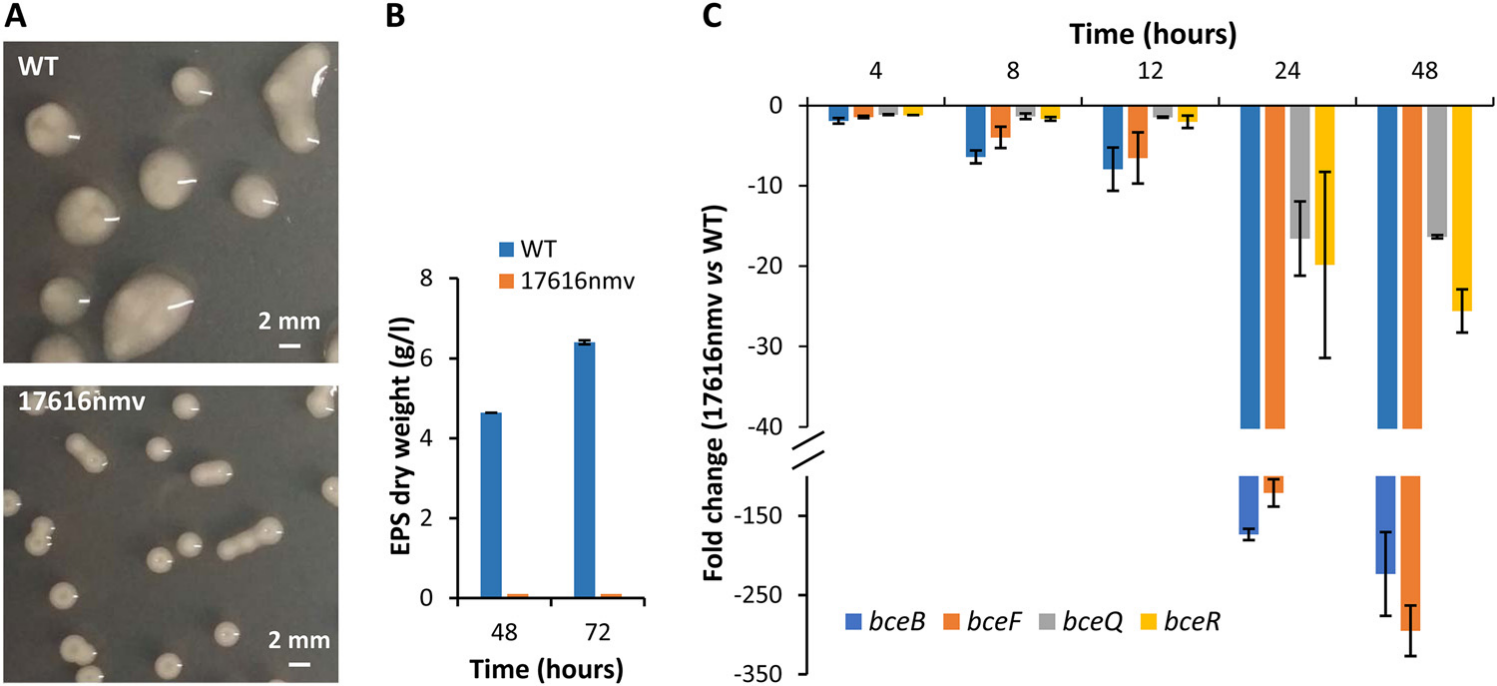
Loss of the mucoid phenotype by a *B. multivorans* ATCC 17616 evolved colony is caused by reduction of *bce* gene expression. (A) Images of the mucoid colony morphologies of the ATCC 17616 ancestor and evolved 17616nmv colony grown in yeast extract-mannitol agar medium for 72 h. (B) Exopolysaccharide production in salts-mannitol medium by the ancestor and evolved colony estimated by recovery of the ethanol precipitate from culture supernatants. (C) Quantitative RT-PCR analysis of transcript levels of *bceB*, *bceF*, *bceQ*, and *bceR* genes in evolved 17616nmv compared to *B. multivorans* ATCC 17616, during growth in salts-mannitol medium up to 48 h at 37°C. Data are the average result from at least three independent growth cultures. Error bars indicate standard deviations. WT, wild type.

We have previously reported the isolation of a nonmucoid variant colony, designated 17616nmv, derived from the mucoid wild-type *B. multivorans* ATCC 17616 after prolonged stationary phase (21 days) in mannitol-salts medium (SM) (22). This phenotypic transition generated smaller colonies when grown in yeast extract-mannitol medium (YEM), while the parental colonies are considerably larger and shiny (Fig. 1A), most likely due to EPS cepacian biosynthesis and secretion. Quantification of the EPS from the broth culture’s supernatants confirmed its absence from the evolved strain’s supernatant, while the ancestor ATCC 17616 produces 4.6 and 6.4 g/liter at 48 and 72 h of growth, respectively (Fig. 1B). To determine whether the absence of cepacian biosynthesis was due to a decreased expression of *bce* genes, the ancestor and evolved strains were grown in SM for 48 h and the expression of genes encoding the priming glycosyltransferase BceB, the tyrosine autokinase BceF, the repeat-unit translocase BceQ, and the bifunctional glycosyltransferase BceR was measured by quantitative real-time PCR (qRT-PCR). Results confirmed the decreased expression of the *bce* genes in the evolved 17616nmv strain (Fig. 1C). Although this decrease was already visible during exponential growth, the highest difference was recorded in the stationary phase at 24 and 48 h. These data indicate that the reduction of the mucoid phenotype of the evolved colony is associated with a strong decrease in the expression of the *bce* genes.

### Genome sequence analysis identified an insertion sequence upstream of a gene encoding a histone-like nucleoid structuring protein.

**FIG 2:**
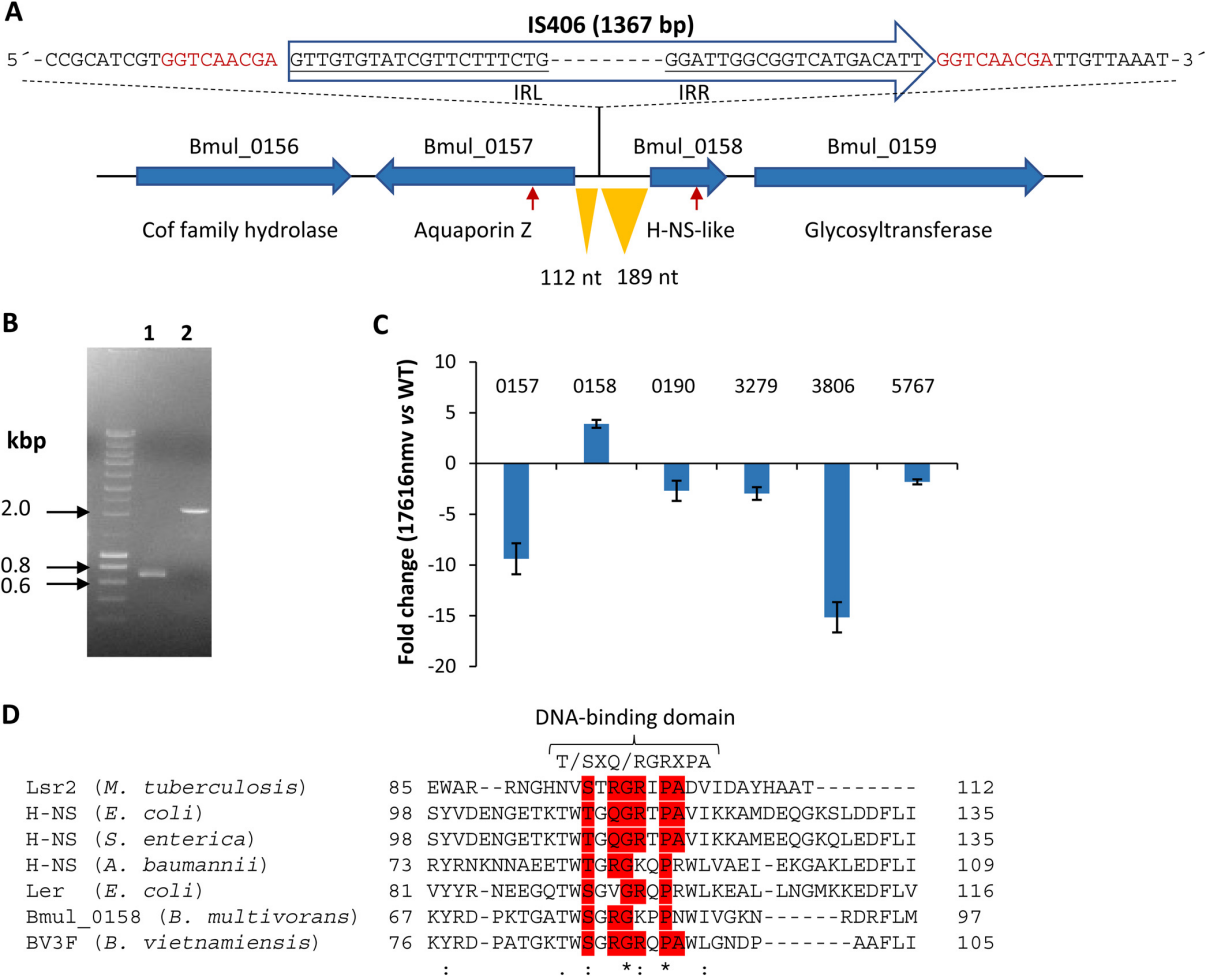
Insertion of the IS*406* element in an intergenic region leads to increased expression of gene Bmul_0158 encoding a histone-like protein. (A) Using whole-genome sequencing of the 17616nmv strain, an insertion sequence of the IS*406* family was identified in the chromosome 1 intergenic region between genes Bmul_0157 and Bmul_0158. The nucleotide sequence of the intergenic region where the IS element was inserted is shown; the target sites of this IS element are shown in red, and partial sequences of the inverted repeats of the IS element are underlined. The two inverted orange triangles indicate the distance from IS*406* to the beginning of Bmul_0157 or Bmul_0158 coding sequences. nt, nucleotides. (B) Electrophoretic separation of the PCR products amplified from the genome of the ancestor ATCC 17616 (lane 1) and the evolved strain 17616nmv (lane 2) with expected sizes of 699 and 2,066 bp, respectively (red arrows in panel A indicate primer-binding sites). (C) qRT-PCR analysis of transcript levels of genes Bmul_0157 and Bmul_0158 and other *hns*-like genes in the evolved strain compared with the ancestor ATCC 17616 grown for 8 h in SM. (D) Amino acid sequence alignments of the C-terminal DNA-binding region of Mycobacterium tuberculosis Lsr2 (ALB20845.1), Escherichia coli K-12 H-NS (NP_415753.1), Salmonella enterica H-NS (AUO51906.1), Acinetobacter baumannii ATCC 17978 H-NS (QDQ68268.1), Burkholderia vietnamiensis Bv3F (A4JS72.1), and *B. multivorans* ATCC 17616 Bmul_0158 (ABX13853) are displayed. The conserved motif T/SXQ/RGRXPA implicated in DNA binding is highlighted in red. Asterisks indicate the amino acid residues that are identical in all proteins; one or two dots indicate semiconserved or conserved substitutions, respectively.

Due to the mucoid phenotypic difference observed between the ancestor and the evolved colony, we sought to sequence the genome of the evolved variant, to identify mutations that could explain the observed difference. The genome was sequenced to a coverage of 65×, and reads were assembled against the *B. multivorans* ATCC 17616 genome sequence present at GenBank. While our analysis did not reveal any single nucleotide variants or small indels, the analysis of reads not fully matching the reference genome allowed the discovery of an insertion sequence (IS) in the chromosome 1 intergenic region between divergently transcribed genes Bmul_0157 and Bmul_0158 (Fig. 2A), showing the same transcription orientation as the Bmul_0158 gene. Bmul_0157 encodes aquaporin Z, and Bmul_0158 encodes a histone-like nucleoid structuring protein. The sequence of this IS element exhibited 100% identity to IS*406* (with a length of 1,367 bp) that, in the ancestor strain genome, is present with six copies in chromosome 1 and one copy in chromosome 3, raising the possibility that the IS element identified within the Bmul_0157/Bmul_0158 intergenic region of the evolved strain was generated by a transposition event. Confirmation of IS*406* insertion at this locus was performed by PCR amplification and Sanger sequencing. As shown in Fig. 2B, amplification of a fragment containing the intergenic region from the ancestor resulted in a fragment of approximately 700 bp, while in the evolved strain, the amplified fragment is approximately 2 kbp (primer binding sites are indicated by red arrows in Fig. 2A).

To analyze whether this IS element affected transcription of genes Bmul_0157 and Bmul_0158, total RNA was extracted from cells collected at late exponential growth phase (8 h) and qRT-PCR was performed (Fig. 2C). Comparison of the expression levels between the evolved strain and the ancestor showed 9.4-fold-decreased expression of the Bmul_0157 gene, possibly by disruption of its promoter, but 3.9-fold-increased expression of Bmul_0158. Such an enhancement of the expression of genes located downstream in the same orientation of IS*406* activation has been reported previously (23), and a similar mechanism is likely responsible for Bmul_0158 increased expression.

To determine whether lack of cepacian biosynthesis in the 17616nmv strain was due to the decreased expression of Bmul_0157 (Fig. 2C), this gene was expressed in *trans* from plasmid pLM20-6. Growth in SM for 72 h did not restore EPS biosynthesis (data not shown), indicating that the nonmucoid phenotype of the variant is likely caused by overexpression of gene Bmul_0158.

The protein encoded by the Bmul_0158 gene is highly conserved between *B. multivorans* strains with genome sequence available, with 100% identity at the amino acid level. The conservation level to other species of *Burkholderia* ranges from 78 to 98%. The overall identity at the amino acid level between the Bmul_0158 gene product and H-NS from other non-*Burkholderia* species is much lower (e.g., percent identity/similarity to Escherichia coli H-NS is 45/60), but there is conservation of the C-terminal region containing the DNA-binding domain (Fig. 2D). The Bmul_0158 amino acid sequence (^78^SGRGKPPN^85^) matches the consensus sequence of this motif almost perfectly (T/SXQ/RGRXPA). Besides Bmul_0158, the genome of *B. multivorans* ATCC encodes additional H-NS-like proteins with lower percent identity/similarity to Bmul_0158. These are Bmul_0190 (44%/66%, respectively) and Bmul_3279 (46/64) located in chromosome 1, Bmul_3806 (52/68) located in chromosome 2, and Bmul_5655 (57/68), Bmul_5701 (37/55), Bmul_5767 (53/68), and Bmul_6044 (34/58) in chromosome 3.

### Overexpression of gene Bmul_0158 in the ancestor abrogates exopolysaccharide biosynthesis.

**FIG 3:**
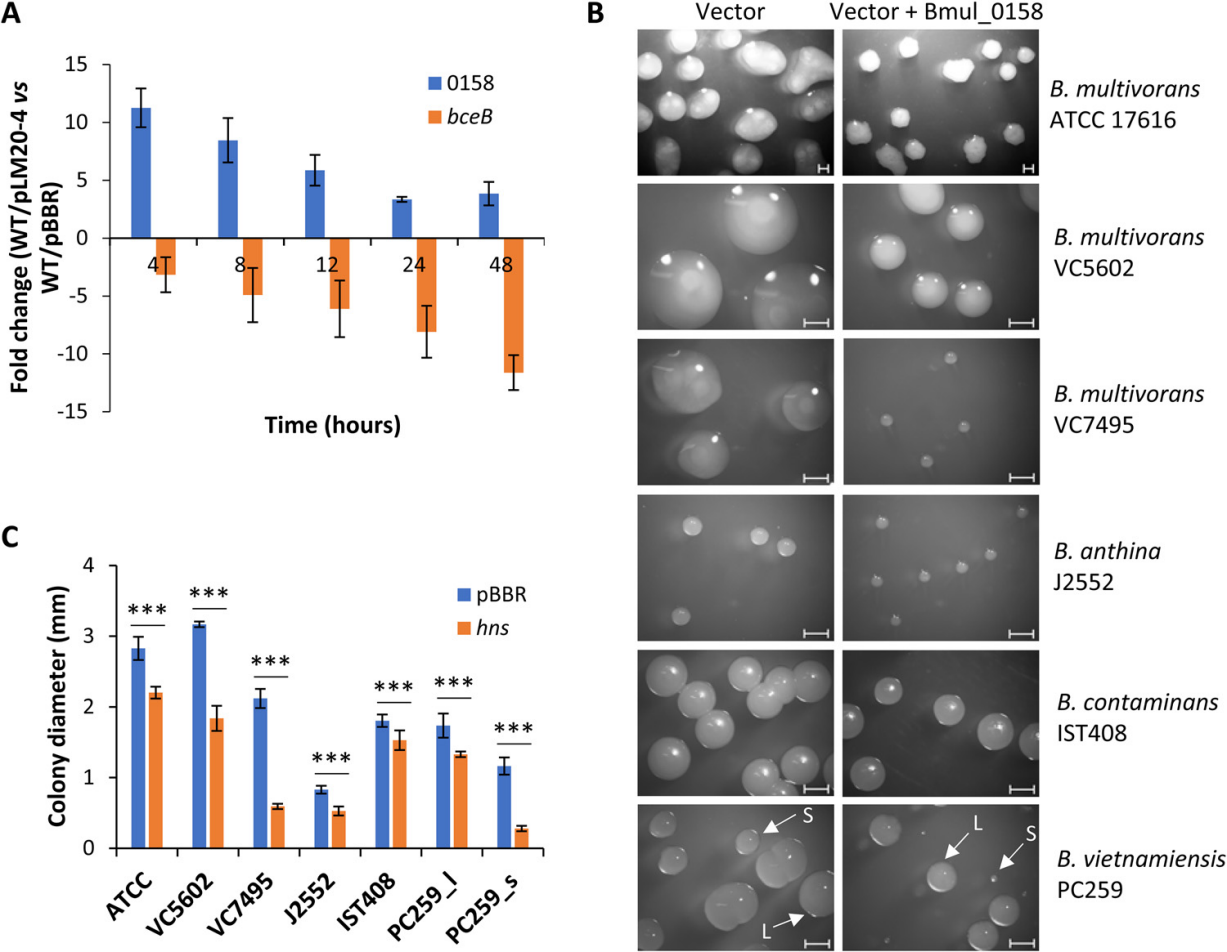
Overexpression of the Bmul_0158 gene in wild-type *Burkholderia* strains reduces the mucoid phenotype of the colonies. (A) qRT-PCR analysis of transcript levels of genes Bmul_0158 and *bceB* in *B. multivorans* ATCC 17616 harboring the vector expressing Bmul_0158 from its own promoter (pLM20-4) compared with ATCC harboring the empty vector pBBR1MCS. (B) Mobilization of the vector (left panel) or pLM20-4 (right panel) to strains of *B. multivorans*, *B. anthina*, *B. contaminans*, and *B. vietnamiensis* followed by incubation in YEM agar medium supplemented with appropriate antibiotics for 3 days. Bar, 1 mm. (C) Quantification of the diameter of at least 20 randomly chosen colonies using the software Zen 3.1 from Zeiss. Colony diameter of strains overexpressing Bmul_0158 was significantly smaller than that for the ones carrying the empty vector. ***, *P* < 0.001 by Tukey’s honestly significant difference (HSD) multiple-comparison test.

To confirm the role of Bmul_0158 H-NS protein in directly or indirectly regulating EPS production, Bmul_0158 coding sequence and its promoter region were cloned into replicative vector pBBR1MCS. This construct, plasmid pLM20-4, was mobilized into *B. multivorans* ATCC 17616 and confirmed by qRT-PCR for the increased expression of the Bmul_0158 gene at all time points compared with the control strain harboring the empty vector (Fig. 3A). To evaluate the effect of Bmul_0158 overexpression on the mucoid phenotype, both strains were grown in yeast extract-mannitol agar medium. As shown in Fig. 3B and C, the overexpression of the Bmul_0158 gene reduced the mucoid phenotype of the colonies (which is also translated into a smaller size of the colonies), while the presence of the empty vector had no effect on the mucoid phenotype. This observation is corroborated by the decreased expression of the *bceB* gene, particularly in stationary phase, in the presence of extra copies of the Bmul_0158 gene (Fig. 3A). We also tested overexpression of the Bmul_0158 gene in other mucoid strains of *B. multivorans* and of other *Burkholderia* species such as B. anthina, *B. contaminans*, and B. vietnamiensis. After 48 to 72 h of incubation in YEM, strains overexpressing Bmul_0158 from the plasmid were unable to develop the mucoid phenotype or presented a reduction in their ability (Fig. 3B and C). Of note, *B. vietnamiensis* PC259 carrying the empty vector displayed heterogeneity in colony sizes, with one type of colony showing an average size of 1.7 mm (Fig. 3B, arrow L, left panel, and Fig. 3C) while the other showed a size of 1.2 mm (Fig. 3B, arrow S, left panel, and Fig. 3C). In the presence of additional copies of Bmul_0158, the average colony sizes decreased to 1.3 and 0.3 mm for L- and S-type colonies, respectively (Fig. 3B, right panel, and Fig. 3C). With these results, we showed the involvement of this H-NS-like protein in regulating a cell envelope phenotype and that this mechanism might be widespread within the genus *Burkholderia*.

### The nonmucoid colony has reduced surface hydrophobicity and forms smaller cell aggregates.

**FIG 4:**
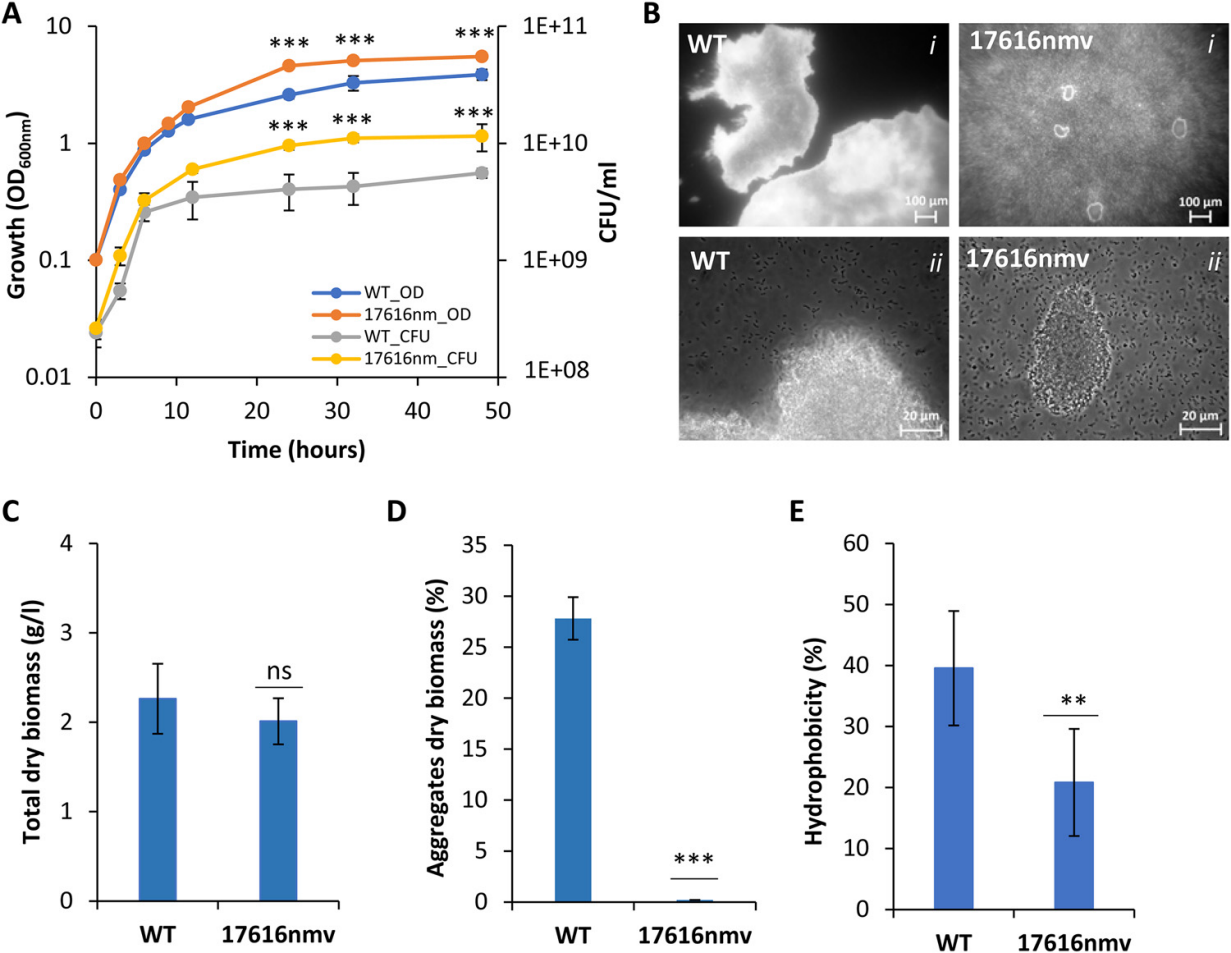
The evolved variant has decreased surface hydrophobicity and forms smaller cellular aggregates. (A) Growth curves of *B. multivorans* ATCC 17616 and evolved 17616nmv in SM at 37°C as measured by optical density and counting of CFU. The evolved variant differed significantly from its ancestor regarding the optical density at 640 nm and CFU at the indicated time points. ***, *P* < 0.001 by Tukey’s HSD multiple-comparison test. (B to D) Light microscopy images of *B. multivorans* ATCC 17616 and evolved 17616nmv grown in SM at 37°C for 48 h (B), followed by dry-weight biomass determination (C) and by quantification of the percentage of aggregates and free cells (D). The evolved variant differed significantly from its ancestor regarding the percentage of cells in the form of aggregates. ***, *P* < 0.001 by Tukey’s HSD multiple-comparison test; ns, nonsignificant. (E) Relative surface hydrophobicity of *B. multivorans* ATCC 17616 and evolved 17616nmv. Bacterial suspensions cultured in SM were adjusted to an OD_640_ of 0.6 (OD_initial_). After the addition of *n*-hexadecane, the OD_aq_ of the aqueous phase was measured. The hydrophobic activity (HP) was calculated from the formula % HP = [1 − (OD_aq_/OD_initial_) × 100]. Values are the means of three independent experiments conducted in triplicates; error bars represent standard deviations. Cell surface hydrophobicity of the evolved variant was significantly lower than that of the ancestor. **, *P* < 0.01 by Tukey’s HSD multiple-comparison test.

To determine whether the overexpression of the Bmul_0158 gene caused by the IS element had an impact on bacterial growth properties, both wild-type ATCC 17616 and the evolved colony were grown in SM, with measurement of the optical density and CFU count (Fig. 4A). The average duplication time based on the two methods was 110 (±5) min for the wild-type strain and 104 (±4) min for the evolved colony, a difference that was not statistically significant. Nevertheless, the biomass of the wild-type strain in the stationary phase, measured by either optical density or CFU count, was consistently lower (*P* < 0.001). Furthermore, visual inspection of broth cultures showed the previously reported large planktonic cellular aggregates for the wild-type strain (24), while no such structures were visible in the evolved variant. Microscopic analysis at two different magnitudes (Fig. 4B, *i* and *ii*) confirms the larger size and denser structure of the wild-type aggregates in comparison to the evolved strain, which showed aggregates smaller than 100 μm. In contrast to the optical density measurement and CFU count, quantification of total dry biomass did not show significant differences between the two strains (Fig. 4C), but the distribution of biomass between aggregates and free cells was different. As shown in Fig. 4D, the percentage of aggregates in the ancestor is 28% while that in the evolved variant is close to 0%. Aggregate formation is most likely the cause for the decrease of optical density and CFU of the wild-type strain reported in Fig. 4A.

To evaluate possible differences at the cell envelope, the surface hydrophobicity of cells in the presence of *n*-hexadecane was measured, with the results showing decreased hydrophobicity for the nonmucoid variant cells (Fig. 4E). Thus, the more hydrophilic cell surface of the variant might cause the lower autoaggregation seen in the evolved variant.

### The nonmucoid variant shows increased motility but decreased adhesion to a CF epithelial cell line.

**FIG 5:**
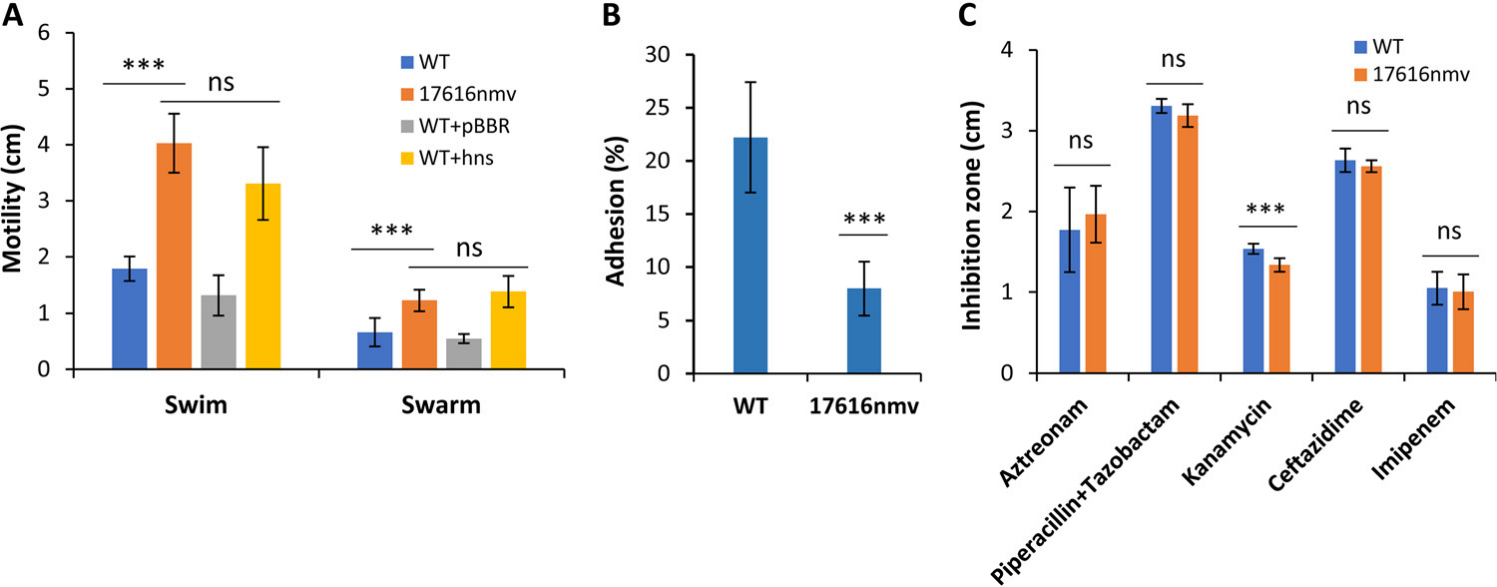
The evolved variant has increased motility but lower adhesion to a CF lung epithelial cell line. (A) Swimming motility assayed in 1% tryptone, 0.5% NaCl medium with 0.3% agar incubated at 37°C for 24 h and swarming motility assayed in Broomfield medium with 0.6% agar incubated at 37°C for 48 h were significantly higher for the strains overexpressing Bmul_0158 compared to the ancestors. ***, *P* < 0.001 by Tukey’s HSD multiple-comparison test. (B) Adhesion to CF lung epithelial cells by *B. multivorans* ATCC 17616 and evolved 17616nmv using an MOI of 10. The evolved variant differed significantly from its ancestor regarding the percentage of cells that adhere to the CF lung cell line. ***, *P* < 0.001 by Tukey’s HSD multiple-comparison test. (C) Susceptibility to the indicated antibiotics determined at 37°C after 24 h of incubation by measuring the diameter of cell growth inhibition. The evolved variant differed significantly from the ancestors only for resistance to kanamycin. ***, *P* < 0.001 by Tukey’s HSD multiple-comparison test.

Literature reports often associate mutations in *hns* genes with alteration in bacterial motility. To evaluate if that also applies in *B. multivorans*, we measured swimming and swarming motilities of the ancestor and evolved variant. We found higher swimming and swarming motilities for the evolved variant compared with the ancestor strain (Fig. 5A). Additionally, when the *hns* gene is overexpressed from a plasmid in the ancestor, this strain showed the same motility trend as the evolved variant.

Due to the decreased ability of autoaggregation by the nonmucoid variant, it is possible that adhesion to biotic surfaces like host cells is compromised. Furthermore, since adhesion to host cells by bacteria is an important virulence mechanism, we evaluated the adhesion of the ancestor and the evolved variant to the cystic fibrosis bronchial cell line CFBE41o- by using a multiplicity of infection (MOI) of 10 bacteria per eukaryotic cell. We found significantly lower adhesion of the evolved variant compared to the ancestor (Fig. 5B), suggesting a role of Bmul_0158 in pathogenesis.

During chronic lung infections of CF patients by *Burkholderia*, the acquisition of bacterial resistance against antibiotics used to treat these infections is common. To determine whether Bmul_0158 could be involved in antimicrobial resistance, we determined the susceptibility of the ancestor and variant against β-lactams ceftazidime, imipenem, aztreonam, and piperacillin plus tazobactam and the aminoglycoside kanamycin. No major differences between ancestor and evolved variant were found, although the variant is slightly more resistant to the antibiotic kanamycin (Fig. 5C).

In conclusion, the increased expression of the *hns*-like gene Bmul_0158 in the evolved variant has no significant impact on antimicrobial resistance but an effect on motility and adhesion to CF bronchial epithelial cells.

### Expression profile confirms phenotypic variability between ancestor and evolved variant.

**FIG 6:**
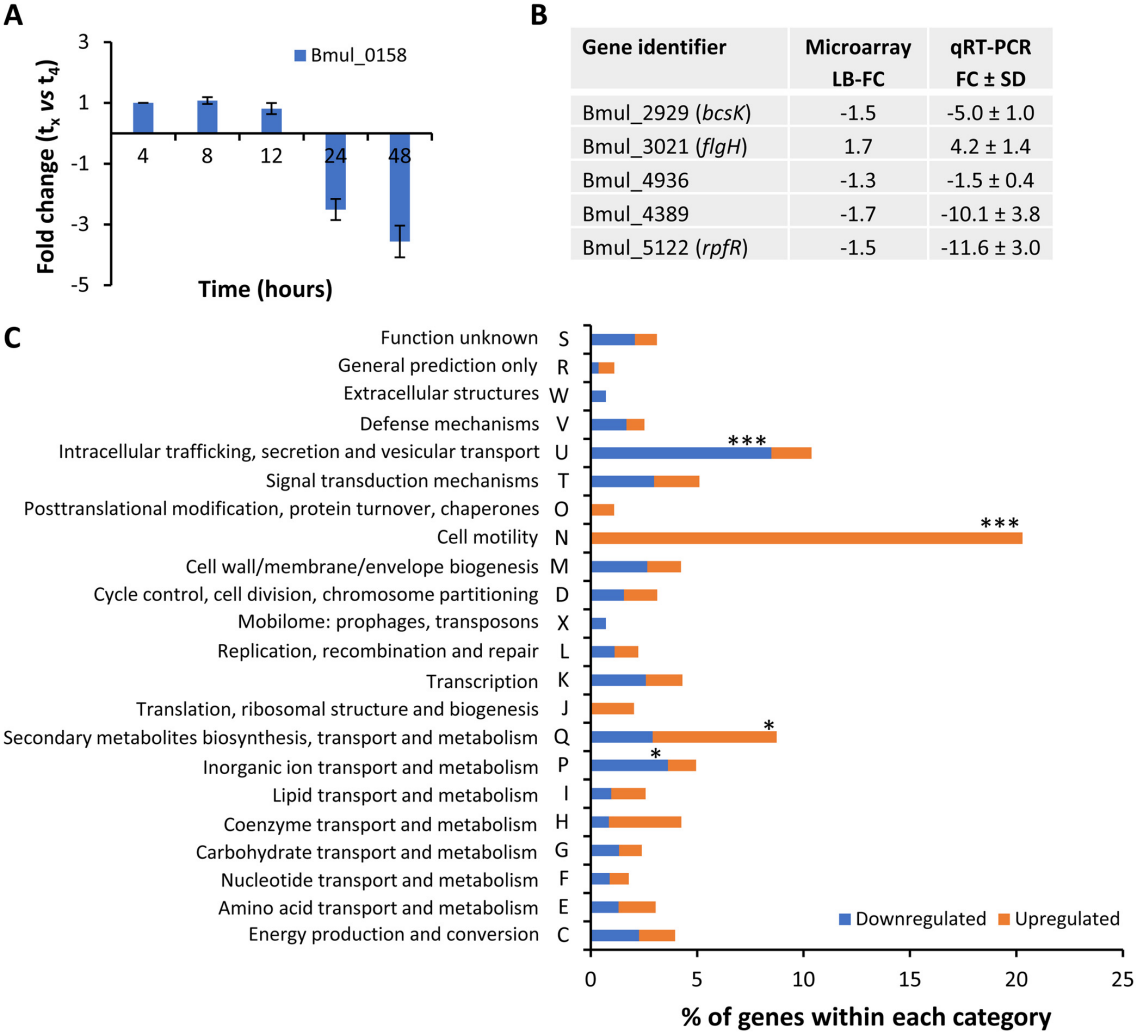
Functional distribution into COGs of genes that are differentially expressed shows enrichment of genes implicated in motility and intracellular trafficking, secretion, and vesicular transport. (A) qRT-PCR analysis of transcript levels of gene Bmul_0158 in *B. multivorans* ATCC 17616 grown in SM. Comparison for the different time points (t_x_) was made against the expression level at 4 h (t_4_). (B) qRT-PCR analysis performed in 17616nmv and *B. multivorans* ATCC 17616 and comparison with the microarray expression data. LB-FC, lower bound of fold change. (C) Clustering, based on biological function, of the differentially expressed genes with COG attributed. The percentage of genes differentially expressed within each category was calculated from the total number of *B. multivorans* genes attributed to each COG category. The cumulative distribution function (CDF) of the hypergeometric distribution was used to calculate enrichment of genes by COG. ***, *P* < 0.001; *, *P < *0.05.

Comparative analysis of the transcriptomes of cells with differences at the genetic level may provide information about the molecular mechanisms and regulatory pathways responsible for certain physiological states. Therefore, we performed whole-genome microarray analysis to examine the effect of overexpression of the *hns*-like gene Bmul_0158 on the transcriptome of *B. multivorans*. For that, the mucoid ancestor and the evolved nonmucoid variant were grown in SM until late exponential phase (10 h of incubation). According to Fig. 6A, this time point is within the period of maximal expression of Bmul_0158, and therefore, it was chosen. A comparison of the expression values of the nonmucoid variant and of the ancestor using a lower confidence interval cutoff for the fold change of 1.2 (false-discovery rate [FDR], 2.4%) showed 133 genes with a significantly decreased expression and 121 genes with a significantly increased expression, which together correspond to 4% of all genes of this genome (the complete list of differentially expressed genes is shown in Table S1 in the supplemental material). To validate the data obtained by microarray analysis, the expression of genes implicated in virulence, motility, transcriptional regulation, and signal transduction was analyzed by qRT-PCR. Results obtained agreed with microarray data, but the fold change for most of them was considerably higher when the expression was analyzed by qRT-PCR (Fig. 6B). Mapping differentially expressed genes to the Clusters of Orthologous Genes (COG) database shows that the enriched categories with upregulated genes are cell motility (11.2-fold over enrichment, *P* < 0.001) and secondary metabolite biosynthesis, transport, and catabolism (3.19-fold over enrichment, *P* < 0.05) (Fig. 6C). Contrastingly, the enriched categories with downregulated genes are intracellular trafficking, secretion, and vesicular transport (4.76-fold over enrichment, *P* < 0.001) and inorganic ion transport and metabolism (2.03-fold over enrichment, *P* < 0.05).

The larger group of genes with significantly increased expression in the nonmucoid variant encode products for flagellum assembly. This comprises gene products involved in the assembly of the motor, several rings, rod, hook, and filament and gene expression regulation (Table 1). Also, genes encoding chemotaxis proteins CheW and CheY and the putative aerotaxis receptor Aer showed increased expression in the variant. This suggests that the nonmucoid variant might have an advantage in environments with limited nutrients and oxygen. The experimental data on swimming and swarming motilities (Fig. 5A) confirm expression data. In opposition to the flagellar system, genes involved in fimbrial protein synthesis and pili assembly (*fimA*, *papC*, and *fimC*) showed decreased expression. Since pili are implicated in adhesion of bacteria to surfaces, this might contribute to the experimentally observed decrease of adhesion to epithelial cells (Fig. 5B). Other genes with decreased expression known to be implicated in adhesion and biofilm formation are Bmul_3907, encoding the large surface protein BapA (25), and the ones involved in the synthesis of a polysaccharide, namely, Bmul_4401 and Bmul_4408, as demonstrated in B. cenocepacia (26).

**TABLE 1:** Selection of a set of genes differentially expressed between 17616nmv and *B. multivorans* ATCC 17616 grown in SM for 10 h, separated by functional groups

Functional class	Gene identifier	Gene name	Description	LB-FC^*a*^
Flagellum synthesis, motility, and chemotaxis	Bmul_0042	*fliN*	Flagellar motor switch protein FliN	1.3
	Bmul_0043	*fliM*	Flagellar motor switch protein FliM	1.2
	Bmul_0044	*fliL*	Flagellar basal body-associated protein FliL	1.2
	Bmul_0151	*fliC*	Flagellin domain-containing protein	2.9
	Bmul_0163	*motB*	Flagellar motor protein MotB	1.3
	Bmul_0164	*cheY*	Response regulator receiver protein	1.4
	Bmul_0166	*cheW*	CheW protein	1.3
	Bmul_0177	*flhF*	Flagellar biosynthesis regulator FlhF	1.2
	Bmul_3009	*flgL*	Flagellar hook-associated protein FlgL	1.3
	Bmul_3016	*flgF*	Flagellar basal-body rod protein FlgF	1.2
	Bmul_3017	*flgE*	Flagellar hook protein FlgE	1.3
	Bmul_3018	*flgD*	Flagellar hook capping protein	1.3
	Bmul_3019	*flgC*	Flagellar basal body rod protein FlgC	1.5
	Bmul_3021	*flgA*	Flagellar basal body P-ring biosynthesis protein	1.7
	Bmul_3022	*flgM*	Anti-sigma-28 factor, FlgM	1.3
	Bmul_3056	*fhlB*	Putative flagellar protein FhlB	1.3
	Bmul_3064	*fliI*	Flagellar protein export ATPase FliI	1.2
	Bmul_3362	*aer*	Putative methyl-accepting chemotaxis sensory transducer with Pas/Pac sensor	1.3
	Bmul_1610	*fimC*	Pilus assembly chaperone	−1.9
	Bmul_1611	*papC*	Fimbrial biogenesis outer membrane usher protein	−1.4
	Bmul_1612	*fimA*	Fimbrial protein	−2.8
	Bmul_1736		Flp/Fap pilin component	−1.8

Chromosome-associated proteins	Bmul_0190		Histone family protein nucleoid structuring protein H-NS	−1.4
	Bmul_2247		Histone deacetylase superfamily	−1.2
	Bmul_3279		Histone family protein nucleoid structuring protein H-NS	−1.3
	Bmul_3806		Histone family protein nucleoid structuring protein H-NS	−1.3
	Bmul_4822	*xseB*	Exodeoxyribonuclease VII, small subunit	−1.3
	Bmul_5655		Histone family protein nucleoid structuring protein H-NS	−1.4
	Bmul_5767		Histone family protein nucleoid structuring protein H-NS	−1.9

Secretion systems	Bmul_2710		Outer membrane autotransporter barrel domain protein	−1.2
	Bmul_4115		Outer membrane autotransporter barrel domain protein	−1.4
	Bmul_2923	*bcsE*	Type VI secretion-associated protein, ImpA family	−1.3
	Bmul_2927	*bcsI*	Type VI secretion system lysozyme-related protein	−1.3
	Bmul_2928	*bcsJ*	Type VI secretion system effector, Hcp1 family	−1.4
	Bmul_2929	*bcsK*	Type VI secretion protein, EvpB/VC_A0108 family	−1.5
	Bmul_2930	*bcsL*	Type VI secretion protein, VC_A0107 family	−1.9
	Bmul_2931	*bcsM*	Type VI secretion protein	−1.5
	Bmul_3709	*bapA*	Hemolysin-type calcium-binding region	−2.4
	Bmul_3907		Type VI secretion protein, VC_A0107 family	−1.2
	Bmul_5730		Type VI secretion system Vgr family protein	−1.4

Transcription regulators	Bmul_1606	*orbS*	RNA polymerase, σ-24 subunit, ECF subfamily	−1.7
	Bmul_2557	*ldhR*	Transcriptional regulator, LysR family	−2.3
	Bmul_4389		Putative transcriptional regulator, Crp/Fnr family	−1.7

Regulatory/signal transduction	Bmul_0879	*glnB*	Nitrogen regulatory protein P-II	−1.5
	Bmul_2393	*cspA*	Cold-shock DNA-binding domain protein	−2.7
	Bmul_3051	*rqpS*	Signal transduction histidine kinase	1.5
	Bmul_3471		Diguanylate phosphodiesterase	−1.4
	Bmul_4947		Response regulator receiver sensor signal transduction histidine kinase	−1.5
	Bmul_5122	*rpfR*	Diguanylate cyclase/phosphodiesterase with PAS/PAC sensor(s)	−1.5

a LB-FC, lower bound of fold change. The lower confidence bound is a conservative estimate of the fold change.

Strongly implicated in phenotypes such as virulence, motility, and biofilm formation are proteins that respond to quorum sensing molecules and to the second messenger cyclic-di-GMP. One of these genes, Bmul_5122, whose expression was decreased in the evolved variant, encodes the *cis*-2-dodecenoic acid (BDSF) receptor RpfR (27). A second gene with decreased expression in the variant is Bmul_4389, encoding an ortholog of BCAM1349 protein from B. cenocepacia, which is a regulator of the cyclic AMP receptor protein (CRP)/fumarate nitrate reductase (FNR) family and is a cyclic-di-GMP effector protein, regulating the expression of genes involved in cellulose biosynthesis and in fimbria assembly (28). In line with the decreased ability of the evolved variant to form cellular aggregates, the LysR-type transcriptional regulator LdhR, regulating the expression of d-lactate dehydrogenase LdhA and implicated in cellular aggregate formation (24), showed decreased expression in the evolved variant (Table 1). Additionally, the decreased expression in the variant of gene *orbS* encoding an extracytoplasmic function sigma factor regulating biosynthesis and transport of the siderophore ornibactin (29) suggests siderophore biosynthesis might also be regulated by H-NS-like proteins.

Type VI secretion system might also be affected in the nonmucoid variant since seven genes implicated in this system showed decreased expression (Table 1). This secretion system is believed to play a role in the maintenance of *Burkholderia* cells in a eukaryotic host by providing protection against other bacteria sharing the same environment (30).

Another set of genes with decreased expression includes five genes encoding H-NS-like proteins, namely, Bmul_0190, Bmul_3279, Bmul_3806, Bmul_5655, and Bmul_5767 (Table 1). As shown in Fig. 2C, qRT-PCR experiments confirmed the decreased expression of the tested *hns*-like genes. This result suggests a possible hierarchy between these *hns*-like genes, with Bmul_0158 being at the top of the regulatory cascade, regulating directly or indirectly a large set of genes, including other *hns*-like genes. Nevertheless, it cannot be excluded that the different *hns*-like genes may cross-regulate the expression of each other’s genes.

### Identification of potential H-NS targets in the ATCC 17616 genome.

**FIG 7:**
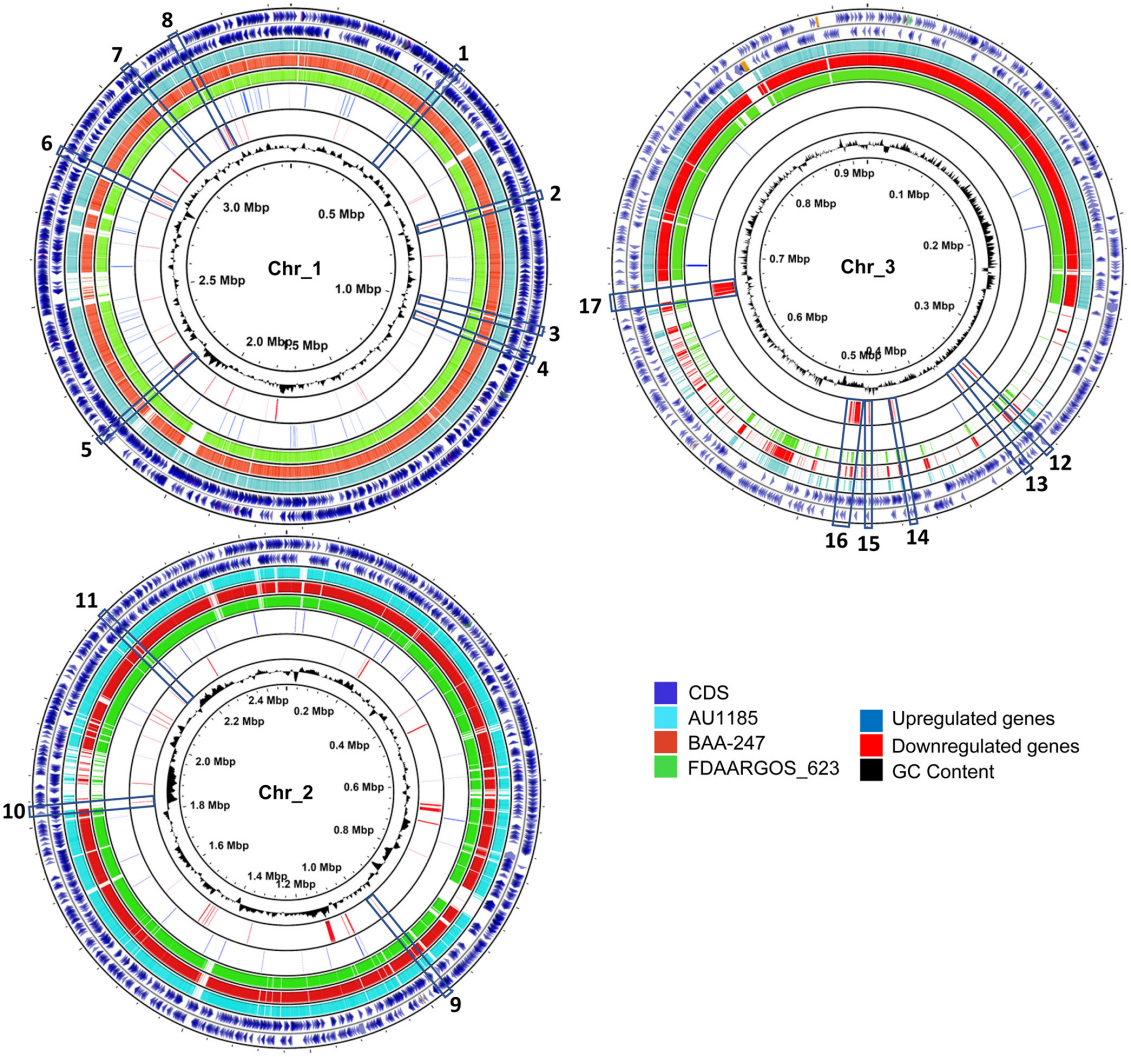
Mapping of the putative Bmul_0158 H-NS protein targets in the ATCC 17616 genome. Gene expression data were mapped onto circular representation of *B. multivorans* ATCC 17616 chromosomes using the software CGView. Downregulated genes are shown in red and upregulated genes in blue, located in the rings between FDAARGOS_623 and the GC content. To identify potentially horizontally acquired genomic regions, comparative BLASTP analyses between ATCC 17616 (outer rings) and *B. multivorans* AU1185 (light blue), FDAARGOS_623 (green), and BAA-247 (red) are included. Various downregulated genes or gene clusters not fully conserved between ATCC 17616 and other genomes were identified (blue boxes), being putative H-NS target sites. CDS, coding sequence.

Horizontally acquired genetic material is often AT rich and is a likely target for H-NS protein regulation. To find these potential regulatory targets of the Bmul_0158 gene product, transcriptomic data were overlaid with the results of BLASTP comparisons of ATCC 17616 with other *B. multivorans* genomes (BAA-247, AU1185, and FDAARGOS_263), on the circular representation of the ATCC 17616 three chromosomes (Fig. 7). Several of the genes with decreased expression and indeed weak conservation among the tested strains are shown by the gaps of BLASTP analysis between *B. multivorans* BAA-247, AU1185, and FDAARGOS_263 genomes and that of ATCC 17616. These 17 potential Bmul_0158 targets include eight regions from chromosome 1, three regions from chromosome 2, and six regions from chromosome 3 (Fig. 7 and Table 2). Chromosome 1 nonconserved downregulated loci include genes putatively involved in the synthesis of a secreted bacterial toxin, a bacterial Toll/interleukin-1-like receptor belonging to proteins known to interfere with the innate immune pathways in the host, a putative O-antigen ligase which might act on lipopolysaccharide biosynthesis, genes putatively involved in bacterial surface antigen activation and secretion and in the biosynthesis of a capsular polysaccharide, and genes putatively involved in type VI secretion. Among the targets of chromosome 2, we identified gene Bmul_5020, encoding a protein putatively binding to colicins to protect their host cells, conferring colicin immunity. Within chromosome 3, some of the genes with decreased expression mapping inside nonconserved regions include gene Bmul_5655, encoding another putative H-NS histone-like protein; Bmul_5730, encoding a type VI secretion-associated protein; and two clusters of genes possibly involved in metabolic pathways. Overall, these analyses suggest that various horizontally acquired genome regions are likely targets for transcriptional repression by the Bmul_0158 gene product in *B. multivorans* and that the increased expression of this H-NS-encoding gene in the variant strain results in downregulation of genes within these regions.

**TABLE 2:** List of putative Bmul_0158 H-NS targets identified in the analysis described in Fig. 7

Region	Locus tag	Gene product or characteristic/function
1	Bmul_0354	Putative secreted bacterial toxin
2	Bmul_0646	Putative phage protein
3	Bmul_0912	Putative bacterial Toll/interleukin-1-like receptor
4	Bmul_0969	Putative O-antigen ligase
5	Bmul_1984 to Bmul_1987	Bacterial surface antigen activation and secretion
6	Bmul_2607, 2608, 2615	Capsular polysaccharide biosynthesis
7	Bmul_2816	Integrase
8	Bmul_2916, 2917	Type VI secretion associated
9	Bmul_3996	Hypothetical protein
10	Bmul_4711, 4712	RNA repair
11	Bmul_5020	Putative colicin binding protein
12	Bmul_5639	Putative dehydrogenase/reductase
13	Bmul_5655	Histone-like protein
14	Bmul_5730	Type VI secretion-associated protein
15	Bmul_5751	Membrane fusion protein of an ABC transporter
16	Bmul_5760 to Bmul_5771	Electron transfer and energy production
17	Bmul_5941 to Bmul_5949	Isoprenoid biosynthesis

**FIG 8:**
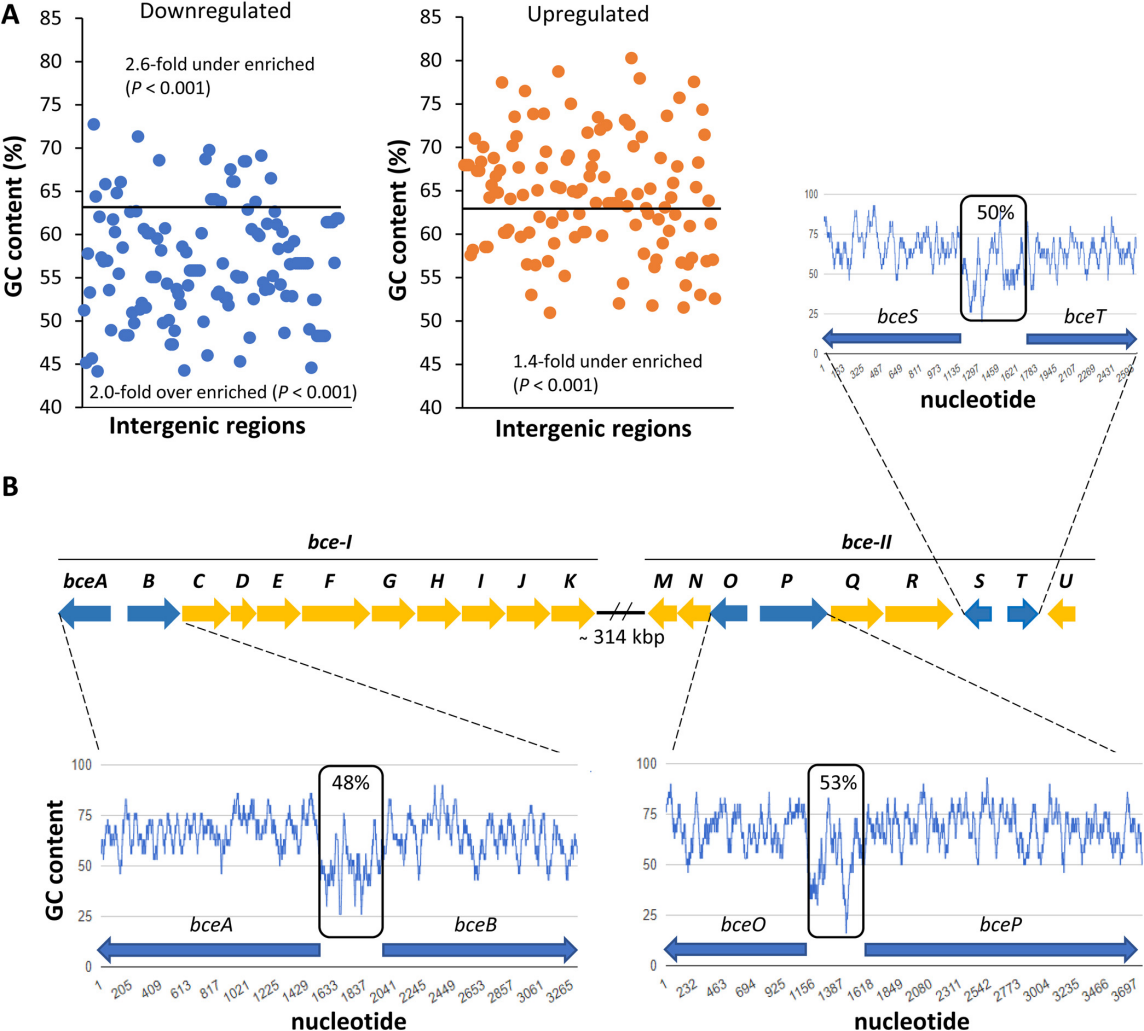
The majority of the downregulated genes have upstream intergenic regions of low GC content. (A) Representation of the GC percentage of the upstream intergenic region of each differentially expressed gene obtained from the microarray data set. The average GC percentage of the *B. multivorans* ATCC 17616 genomic-wide intergenic regions was determined as being 63.4%. The cumulative distribution function of the hypergeometric distribution was used to calculate enrichment of genes above or below 63.4% GC content. (B) Schematic representation of the *bce* gene clusters directing the biosynthesis of cepacian, with indication of the GC percentage of three intergenic regions containing putative promoters for genes *bceA* and *bceB* to -*K*, *bceO* to -*M*, *bceP* to -*R*, and *bceS*-*T* as indicated by black boxes. The GC content plots were derived from the *B. multivorans* ATCC 17616 genome using the GC content calculator at Biologics International Corp.

The analysis of the global GC content shown in the three replicons reveals that several downregulated genes are in loci of high-AT-percentage regions (Fig. 7), as expected from the ability of H-NS proteins to bind preferentially to such regions. Additionally, when examining the upstream regions of the differentially expressed genes for their GC content, we observed a 2-fold overenrichment (*P* < 0.001) of downregulated genes with a GC content below the average GC percentage of the ATCC 17616 genome-wide intergenic regions, which is 63.4% (Fig. 8A). Contrastingly, the downregulated genes with an upstream intergenic region with a GC content above 63.4% were 2.6-fold underenriched (*P* < 0.001). In the group of upregulated genes, we observed 1.4-fold underenrichment (*P* < 0.001) of the ones with a GC content of the upstream intergenic region below 63.4%, while the number of the ones above that value was the expected one (Fig. 8A). This analysis highlights an enrichment of low-GC-content promoters as possible targets of Bmul_0158. As an example, the inspection of the GC content of two promoter regions within the *bce* gene clusters shows a GC content between 48 and 53% (Fig. 8B) with stretches of AT-rich regions that could be binding sites for H-NS proteins.

## DISCUSSION

This study reports the effect of an IS element on the overexpression of an H-NS-like protein from *B. multivorans* with impact on regulating gene expression and phenotypic traits implicated in persistence and virulence, such as the biosynthesis of the exopolysaccharide, cellular aggregation, adhesion to lung epithelial cells, and motility. To survive and adapt to environmental changes, bacteria face major challenges in packing their DNA and controlling gene expression. A strategy for that is the use of various nucleoid-associated proteins (31, 32). Nucleoid-associated H-NS proteins have been characterized in several bacteria and are known to regulate the expression of genes that ultimately affect traits such as biofilm formation, adhesion to biotic surfaces, motility, exopolysaccharide biosynthesis, and antimicrobial resistance, among others (33). Nevertheless, their implication in a certain trait varies according to the regulatory circuits of that microorganism. As an example, the deletion of the *hns* gene of Vibrio cholerae increased the expression of genes involved in EPS production (18), while in Vibrio parahaemolyticus the *hns* mutant shows decreased expression of genes involved in EPS biosynthesis (20). Here, we show that an increased expression of the Bmul_0158 gene encoding an H-NS-like protein strongly decreased the expression of the *bce* genes, with no concomitant production of cepacian exopolysaccharide. Three mechanisms can be proposed for the role of this *B. multivorans* H-NS-like protein in silencing *bce* gene expression. In the first, a larger amount of H-NS protein would bind to the promoters of the two *bce* gene regions, inhibiting the binding of transcription factors to these regions and consequently preventing the activation of RNA polymerase. In the second mechanism, H-NS would decrease the expression of genes encoding transcriptional regulators controlling *bce* gene expression. The third mechanism is a combination of the two in which repression of *bce* gene expression could be achieved by lowering the number of regulatory proteins and by increasing H-NS proteins at the promoter regions of those *bce* genes. So far, only two response regulators, OmpR and NtrC, from the signal transduction systems EnvZ/OmpR and NtrB/NtrC, respectively, have been implicated in the regulation of cepacian biosynthesis in *Burkholderia* (12, 13, 34), but it is unknown whether these regulators bind directly to the *bce* gene regions or not. The expression data presented here show that neither *ompR* nor *ntrC* genes are differentially expressed in 17616nmv compared with the wild-type strain, suggesting that repression by Bmul_0158 H-NS-like protein at the *bce* promoters is the most likely mechanism for cepacian biosynthesis regulation. This is also supported by the expression of Bmul_0158, which is reduced during the stationary phase (Fig. 6A), promoting *bce* genes’ expression. In line with this is the low GC content of three promoter regions within the *bce* gene clusters that could be binding sites for H-NS proteins (Fig. 8B). Structurally, H-NS proteins have an N-terminal oligomerization domain, a C-terminal DNA-binding domain, and an unstructured linker region. Assembly of protein-DNA complexes is believed to proceed via a multistep process in which the C-terminal DNA-binding domain directs the H-NS protein to a high-affinity site in DNA which may serve as a nucleation site for subsequent polymerization on lower-affinity AT-rich sites. Then, H-NS proteins would spread cooperatively along the DNA, forming a nucleoprotein filament by oligomerization through their N-terminal domains. If the conditions are favorable, these nucleoprotein filaments can interact with another DNA duplex to form a bridge (35, 36). H-NS-DNA filament formation (either nucleoprotein filaments or bridged complexes) at or across a promoter region potentially blocks RNA polymerase, preventing the initiation of gene transcription. Similar mechanisms could be acting at the *bce* gene promoters.

Motility is another trait that has been described as being dependent on H-NS proteins. In Acinetobacter baumannii ATCC 17978, Mycobacterium smegmatis, and Shewanella piezotolerans, the disruption of the *hns* gene caused increased motility (37–39). In contrast, in *B. multivorans* the observed increased motility is caused by the increased expression of Bmul_0158, encoding an H-NS-like protein. The RqpSR two-component regulatory system of B. cenocepacia has been demonstrated to exert a positive effect upon the quorum sensing system *cis*-2-dodecenoic acid and *N*-acylhomoserine lactone synthase gene promoters and therefore to affect phenotypes such as motility, biofilm formation, and virulence (40). Since the gene *rqpS* shows increased expression in the 17616nmv strain, it could perhaps affect motility positively by a quorum sensing-dependent manner or directly through the response regulator RqpR.

In E. coli, the H-NS protein was found to be a global regulator of gene expression affecting 5 to 10% of the genes, mostly by repression (41). MvaT regulates hundreds of genes in Pseudomonas aeruginosa, and Lsr2, another H-NS protein, binds to one-fifth of the Mycobacterium tuberculosis genome, especially to horizontally acquired genes (42, 43). In contrast, in A. pleuropneumoniae, H-NS does not act as a global gene regulator but rather specifically regulates the poly-β-1,6-*N*-acetyl-d-glucosamine (19). According to our expression data, Bmul_0158-encoded H-NS protein seems to be a global regulator, acting slightly more as a repressor. Regarding the targets of H-NS proteins, they are mainly horizontally acquired AT-rich genetic material (16). Some examples of horizontally acquired genes are those involved in type III and type VI secretion systems in V. parahaemolyticus (44) and genes involved in type VI secretion, autotransporter adhesin Ata, and an *S*-adenosyl-l-methionine-dependent methyltransferase in A. baumannii (37). As evidenced by mapping of the 17616nmv strain downregulated genes into the genomes of three other strains of *B. multivorans*, a good number of them are absent in these genomes, implying they are most likely acquired by horizontal gene transfer and are targets for transcriptional repression by the Bmul_0158-encoded H-NS protein. Of special relevance due to their possible role in pathogenicity are genes encoding the type VI secretion system, immunogenic proteins, putative bacteriocins, and proteins related to interaction with the host. Additionally, the low GC content of genes possibly acquired by horizontal transfer (Fig. 7) points to a correlation between transcriptional downregulation and high AT content.

Understanding the regulatory circuits dependent on the H-NS-like family of nucleoid structuring proteins is a challenge, especially when several genes encoding H-NS-like proteins are present. In contrast to E. coli or Salmonella, each of which has one to two *hns*-like genes, *Burkholderia* strains have multiple copies distributed between chromosomes and plasmids. The existence of more than one H-NS-like protein in the same microorganism might represent a partition of function between the different proteins. Moreover, they might form oligomers, providing an additional level of complexity between the heteromeric protein interactions and DNA (33). Transcriptomic data show that Bmul_0158 overexpression in the variant strain decreases the expression of five out of seven *hns*-like genes. Whether the Bmul_0158 H-NS protein is the major H-NS-like protein in *B. multivorans* or whether functional divergence between them exists is unknown, but the way these different H-NS proteins regulate each other is certainly worth pursuing.

Given the strong effects of fluctuations in Bmul_0158 *hns*-like gene expression level on several traits related to persistence and virulence of *Burkholderia* in the CF lung, understanding the mechanisms by which H-NS proteins regulate gene expression may provide new insight into *Burkholderia* virulence.

## MATERIALS AND METHODS

### Bacterial strains and growth conditions.

The bacterial strains and plasmids used are described in Table 3. *Burkholderia* and E. coli strains were routinely grown in Lennox broth (LB) at 37°C, supplemented with antibiotics when appropriate. Antibiotics were used at the following concentrations for *B. multivorans*: chloramphenicol, 200 μg/ml; and gentamicin, 40 μg/ml; for E. coli, concentrations were as follows: chloramphenicol, 25 μg/ml, and kanamycin, 50 μg/ml. Growth in liquid medium was analyzed in triplicates through measurement of the optical density at 640 nm (OD_640_) over time. Depending on the phenotype test, *Burkholderia* strains were grown in salts-mannitol medium (SM) (12.5 g/liter Na_2_HPO_4_, 3.0 g/liter KH_2_PO_4_, 1.0 g/liter K_2_SO_4_, 1.0 g/liter NaCl, 0.2 g/liter MgSO_4_·7H_2_O, 0.001 g/liter CaCl_2_·2H_2_O, 0.001 g/liter FeSO_4_·7H_2_O, 1.0 g/liter Casamino Acids, 1.0 g/liter yeast extract, 20 g/liter d-mannitol) or YEM (0.5 g/liter yeast extract, 4 g/liter d-mannitol, 2% agar).

**TABLE 3:** Strains and plasmids used in this work

Strain or plasmid	Relevant characteristic(s)^*a*^	Reference or source
Strains		
*Burkholderia*		
*B. multivorans* ATCC 17616	Soil isolate, USA; EPS^+^	60
*B. multivorans* ATCC 17616nmv	Nonmucoid variant obtained from ATCC 17616 under nutrient starvation for 21 days	22
*B. multivorans* VC5602	Cystic fibrosis isolate, Canada; EPS^+^	59
*B. multivorans* VC7495	Cystic fibrosis isolate, Canada; EPS^+^	D. P. Speert
*B. contaminans* IST408	Cystic fibrosis clinical isolate, Portugal; EPS^+^	61
*B. anthina* J2552	Rhizosphere, United Kingdom; EPS^+^	62
*B. vietnamiensis* PC259	Cystic fibrosis isolate, USA; EPS^+^	63
Escherichia coli DH5α	DH5α *recA1* Δ(*lacZYA-argF*)*U169* Φ80d*lacZ*ΔM15	Gibco BRL

Plasmids		
pRK2013	Tra^+^ Mob^+^ (RK2) Km::Tn*7* ColE1 origin, helper plasmid, Km^r^	64
pBBR1MCS	4,717-bp broad-host-range cloning vector, Cm^r^	65
pLM20-4	pBBR1MCS derivative containing a 579-bp fragment with the coding sequence of Bmul_0158 and its upstream region	This work
pLM20-6	pBBR1MCS derivative containing a 1,072-bp fragment with the coding sequence of Bmul_0157 and its upstream region	This work

a EPS^+^, exopolysaccharide producer; Cm^r^, chloramphenicol resistance; Km^r^, kanamycin resistance.

### DNA manipulation and cell transformation.

Genomic DNA from *Burkholderia* was extracted by using the DNeasy blood and tissue kit (Qiagen) according to the manufacturer’s recommendations. Plasmid DNA isolation and purification, DNA restriction, agarose gel electrophoresis, DNA amplification by PCR, and E. coli transformation were performed using standard procedures (45). Triparental conjugation to *Burkholderia* strains was performed using the helper plasmid pRK2013.

### Whole-genome sequence determination and *in silico* mutation analysis.

The DNA extracted from the evolved 17616nmv was processed according to Illumina instructions for library preparation and sequenced using an Illumina MiSeq system at Instituto Gulbenkian de Ciência (Portugal). Raw paired-end reads of 2× 250 bp were filtered on the basis of Phred quality scores, with adapter contamination and ambiguous nucleotides trimmed using the fastq-mcf tool (46). The filtered paired-end data set was mapped against the reference genome sequence of *B. multivorans* ATCC 17616 available at GenBank using BWA-MEM v0.7.17 (47), and the detection of mutations was carried out using SAMtools v0.1.19 (48). Predicted mutations were manually inspected using Geneious v6.1.8 (49).

### Presence of the IS*406* element in the 17616nmv genome.

Experimental confirmation of the insertion of the IS*406* element in the genome of the 17616nmv strain was done by amplification of a product with an expected size of 699 bp if IS*406* is absent and 2,066 bp if it is present. Primers used were Bmul_0157/0158_IG_Fw and Bmul_0157/0158_IG-Rev (Table 4). Amplification conditions were an initial denaturation of 1 min at 94°C; 30 cycles of 30 s at 94°C, 30 s at 56°C, and 100 s at 72°C; and a final extension of 7 min at 72°C.

**TABLE 4:** List of primers used in this work

Primer set	Sequence forward^*a*^	Sequence reverse^*a*^
Bmul_0157/0158_IG	CAGATGGCAGCCCGAAATGTG	GTCTTCGGATCGCGGTATTTCG
Bmul_0158	GGGTACCTAGGAAATGAAACGCCGCATCG	CTCTAGAAATCCGGCGCGTAAAACCAAAG
Bmul_0157	GGGTACCTCCCCTTTCCATGATGCGCCCCG	CTAGTCTAGATGCTCTGCTCGCATGACCCCG
qRT-Bmul_0157	ACCGGAATCGAGATCAGGTG	GTGATGACGGGCTTCTTCCT
qRT-Bmul_0158	AACTCAAGGCCCAGATTGCC	GTCAGCCCGTATTCGTCGAT
qRT-Bmul_0190	GCAGAAAATCGCCGACTACG	GTATTTTGCCGCCACACCG
qRT-Bmul_3279	CGTGCAACTCGAAAGGCTTC	AGCTCTTCCGGCGTAATGTC
qRT-Bmul_3806	GCCGTCCGAAAATCTGTTCC	TGCAAAAAGAAGCGGAAGCC
qRT-Bmul_5767	CTTTGCTTCAGGTTGCACGG	GCAAGATCGCAGAGTACGGT
qRT-bceB	TTCGTGAACATCCGCTTCATT	CCGAGCACCTCGACCACTT
qRT-bceF	AACACTCCTACGCGGATCTGT	CAGCCAGATGTCGTCCATGA
qRT-bceQ	GGACAAAGGCATACTCAAGAACGT	CGAAGGTCGGCAGGATCA
qRT-bceR	GTGTCCCAGTTGCGGATGTA	TGGAACAGTCGAACCTCACG
qRT-proC	GTCGGCGAGATCGTAGGTT	CTGCAGCGCTTCGATGAAA
qRT-sigE	GAGATGAGCACCGATCACAC	CCTTCGAGGAACGACTTCAG

a Restriction sites are underlined.

### Cloning of Bmul_0157 or Bmul_0158 coding sequence and upstream region.

A 579-bp fragment containing the Bmul_0158 coding sequence and the upstream promoter region was PCR amplified from *B. multivorans* ATCC 17616 genomic DNA, with Bmul_0158 forward and reverse primers (Table 4). For cloning Bmul_0157 and its upstream region, primers Bmul_0157 forward and reverse were used to amplify a 1,072-bp fragment. The obtained KpnI-XbaI fragments were then cloned into pBBR1MCS digested with the same enzymes to generate pLM20-4 and pLM20-6 containing Bmul_0158 and Bmul_0157, respectively (Table 3). These plasmids (or the empty vector) were mobilized from E. coli to *Burkholderia* strains by triparental conjugation. Transconjugants were selected on LB or YEM agar plates containing 200 μg/ml of chloramphenicol and 40 μg/ml of gentamicin.

### *In silico* analysis of amino acid sequences.

The algorithm BLAST (50) was used to compare sequences of Bmul_0158 amino acids to database sequences available at the National Center for Biotechnology Information (NCBI). Alignments were performed using the program Clustal Omega (51).

### Isolation of RNA samples.

For expression profiling by using microarrays, overnight cultures of *B. multivorans* ATCC 17616 and 17616nmv grown in SM were diluted to an initial OD_640_ of 0.1 into the same medium and cultured at 37°C, 180 rpm, for 10 h. To measure the expression levels of *hns*-like genes and *bce* genes by qRT-PCR, cells were grown under the above conditions for 8 and up to 48 h, respectively. Three biological replicates were obtained for each tested strain. For RNA analysis, bacterial cells were resuspended in RNAprotect bacterial reagent (Qiagen), and total RNA extraction and purification were carried out using the RNeasy minikit (Qiagen), followed by treatment with DNase for 1 h at 37°C according to the manufacturer’s protocol. Total RNA concentration was assessed using a NanoDrop ND-1000 spectrophotometer.

### qRT-PCR.

For the reverse transcription reaction, 1 μg of total RNA from the strains under study, derived from three independent samples, was used. cDNA was synthesized using TaqMan reverse transcription reagents (Applied Biosystems) according to the manufacturer’s instructions. The primers used to amplify the chosen genes (Table 4) were designed using Primer Express 3.0 software (Applied Biosystems). qRT-PCR amplification mixtures used 400 ng of template cDNA, 2× SYBR green PCR master mix, and 0.4 mM reverse and forward primers for each gene, in a total volume of 25 μl. Reactions were performed with a model 7500 thermocycler from Applied Biosystems. The expression ratio of the target genes relative to the reference gene *proC* or *sigE*, which showed no variation in transcription abundance under the conditions tested, was determined. Relative quantification of gene expression was determined using the threshold cycle (ΔΔ*C_T_*) method (52).

### Processing of RNA samples for microarray analysis.

RNA integrity was checked on an Agilent 2100 Bioanalyzer using an RNA Nano assay (Agilent Technologies). RNA was processed for use on Affymetrix (Santa Clara, CA, USA) custom dual-species *Burkholderia* arrays, as previously described (53).

### Microarray analysis.

Scanned arrays were analyzed with Affymetrix Expression Console software to ensure that all quality parameters were in the recommended range. Subsequent analysis was carried out with DNA-Chip Analyzer 2008. First, a digital mask was applied, leaving for analysis only the 9,610 probe sets on the array representing Burkholderia multivorans ATCC 17616 transcripts. Then, the 6 arrays were normalized to a baseline array with median CEL intensity by applying an invariant set normalization method (54). Normalized CEL intensities of the arrays were used to obtain model-based gene expression indices. Replicate data (triplicates) for each strain were weighted gene-wise by using inverse squared standard error as weights. All genes compared were considered differentially expressed if the 90% lower confidence bound of the fold change between experiment and baseline was above 1.2, resulting in 254 differentially expressed transcripts with a median false-discovery rate (FDR) of 2.4%. The lower confidence bound criterion means that we can be 90% confident that the fold change is a value between the lower confidence bound and a variable upper confidence bound. The lower confidence bound is a conservative estimate of the fold change and therefore more reliable as a ranking statistic for changes in gene expression (54).

### Mapping of expression data to *B. multivorans* genomes.

The CGView server (55) was used to compare the genome of *B. multivorans* ATCC 17616 to the ones of *B. multivorans* AU1185, BAA-247, and FDAARGOS_623, through BLASTP analysis. Then, genes differentially expressed were mapped against ATCC 17616 coding sequence (CDS) to identify regions possibly acquired by horizontal gene transfer.

### Determination of growth rates.

Cultures were grown in SM at 37°C with 180-rpm orbital agitation, and the OD_640_ was measured for 48 h. Simultaneously, CFU were also determined by plating serial dilutions into LB solid medium. Three independent experiments were performed.

### Exopolysaccharide quantification.

Exopolysaccharide production was assessed based on the dry weight of the ethanol-precipitated polysaccharide recovered from 100-ml culture samples of the two strains grown in liquid SM for 3 days at 37°C with orbital agitation as previously described (56).

### Quantification of cellular aggregates and free cells.

Cellular aggregates and free cells were quantified according to a previously described protocol (57), with a few modifications. After growing mutant cultures for 48 h in SM at 37°C and 180-rpm orbital agitation, the content of each Erlenmeyer flask was transferred to a 50-ml Falcon tube, which was then centrifuged at 2,000 × *g*, 25°C, for 30 s. The supernatant obtained was transferred to a second 50-ml Falcon tube, and the loose pellet was transferred to a 2-ml Eppendorf tube, previously weighed. This was subjected to short-spin centrifugations, and the resulting pellet, corresponding to cellular aggregates, was obtained. The Falcon tube with the free cells’ supernatant was centrifuged for 10 min at 8,000 × *g*, 25°C. Cell sediment was transferred to a 2-ml Eppendorf tube, also previously weighed. Both Eppendorf tubes of cellular aggregates and free cells were left to dry at 60°C for 72 h and weighed again. Results are the means from data from three independent experiments.

### Bacterial surface hydrophobicity.

The relative surface hydrophobicity of the *B. multivorans* ancestor and the evolved variant was determined by measuring their ability to absorb *n*-hexadecane as described previously (58). Briefly, bacterial cells grown in SM were washed twice in phosphate-buffered saline (PBS) (10 mM Na_2_HPO_4_, 1.8 mM KH_2_PO_4_, 0.14 M NaCl, 2.7 mM KCl, pH 7.3) by centrifugation at 10,000 × *g* for 5 min and resuspended in the same buffer. Bacterial suspensions were adjusted to an optical density of 0.6 (6 × 10^8^ cells/ml) at OD_640_ (OD_initial_). To one and one-half milliliters of this suspension, 400 ml of *n*-hexadecane (Sigma Chemical Co.) was added. This mixture was vortexed for 30 s and then incubated for 30 min at room temperature. The optical density of the cell suspensions after (OD_aq_) addition of *n*-hexadecane was measured at 640 nm, and the hydrophobicity index (HI), expressed as a percentage, was calculated using the following formula: HI (%) = [1 − (OD_aq_/OD_initial_) × 100]. Experiments were performed at least three times.

### Motility.

Swimming and swarming motilities were carried out as described previously (59). Swimming agar plates contained 1% (wt/vol) tryptone, 0.5% (wt/vol) NaCl, and 0.3% (wt/vol) Noble agar (Difco). Swarming plates contained Broomfield medium (0.04% [wt/vol] tryptone, 0.01% [wt/vol] yeast extract, 0.0067% [wt/vol] CaCl_2_) with 0.6% (wt/vol) Bacto agar (Difco). Agar plates were spot inoculated with a 5-μl drop of a culture at an OD_640_ of 1.0. After inoculation, swimming plates were incubated for 24 h at 37°C while swarming plates were incubated for 48 h. Following that period, the diameter of the swarming and swimming zones was measured. Results are the means from data from at least 10 replicates of two independent experiments.

### Antimicrobial susceptibility and zone inhibition assays.

Antimicrobial susceptibility tests were based on the agar disc diffusion method against piperacillin (75 μg) plus tazobactam (10 μg), ceftazidime (30 μg), kanamycin (30 μg), imipenem (10 μg), and aztreonam (30 μg). The discs, obtained from Becton, Dickinson, were applied onto the surface of Mueller-Hinton agar plates (Difco Laboratories) that had been previously inoculated with 100-μl aliquots prepared from cultures grown overnight in LB, at 37°C, with agitation, and diluted to a standardized culture OD_640_ of 0.1. The diameters of growth inhibition were measured after 24 h of incubation at 37°C. Results are the mean values from at least three independent determinations with four replicates each.

### Host cell attachment.

*B. multivorans* isolates were analyzed for adhesion to the bronchial epithelial cell line CFBE41o-, derived from a patient homozygous for the cystic fibrosis transmembrane conductance regulator F508del mutation. Bacterial strains were grown overnight, after which 200 μl of those cultures was grown in LB for 4 h and then used to infect epithelial cells at a multiplicity of infection (MOI) of 10 (10 bacterial cells to 1 epithelial cell). Bacteria were applied to a 24-well plate previously seeded with CFBE41o- cells in supplemented minimal essential medium, and plates were centrifuged at 700 × *g* for 5 min. Plates were then incubated for 30 min at 37°C in an atmosphere of 5% CO_2_. Afterward, each well was washed three times with PBS to remove unbound bacteria, and cells were lysed with lysis buffer (0.01 M PBS, 10 mM EDTA, 0.25% [vol/vol] Triton X-100, pH 7.4) for 20 min at 4°C. Serial dilutions were plated on LB agar, and adhesion was quantified by determining CFU counts after 48 h of incubation at 37°C. Duplicates with each strain were performed per assay, and the results presented were obtained from three independent experiments. Results are shown as the percentage of adhesion, which was calculated as the number of CFU recovered divided by the number of CFU applied to the epithelial cells multiplied by 100.

### Statistical analysis.

Error propagation was used to calculate standard errors, and one-way analysis of variance (ANOVA) and Tukey’s multiple-comparison test were performed to assess statistical significance. Enrichment analysis was performed based on the cumulative distribution function of the hypergeometric distribution. Differences were considered statistically significant if the *P* value was lower than 0.05.

### Accession number.

Microarray data were deposited in the Gene Expression Omnibus (GEO) repository under accession number GSE162693.
